# Electric Properties Change during Morphological Evolution of CeO_2_ Nanostructures: Synergy between Bulk and Surface Defects

**DOI:** 10.1021/acsomega.4c03210

**Published:** 2024-10-03

**Authors:** Carlos Macchi, Alley M. S. Procópio, Leandro S. R. Rocha, Pedro P. Ortega, Celso M. Aldao, Luis I. Granone, Hugo M. S. Nascimento, Elson Longo, Miriam S. Castro, Fernando D. Ivorra, Alberto Somoza, Francisco Moura, Miguel A. Ponce

**Affiliations:** † CIFICEN (UNCPBA-CICPBA-CONICET) and Materials Physics Institute (IFIMAT), National University of Central Buenos Aires (UNCPBA), Tandil 7000, Argentina; ‡ Advanced Materials Interdisciplinary Laboratory (LIMAv), Federal University of Itajubá, Itabira, Minas Gerais 35903-087, Brazil; § Materials Engineering Department, Federal University of São Carlos (UFSCar), São Carlos, São Paulo 13565-905, Brazil; ∥ Center for Research and Development of Functional Materials (CDMF), Federal University of São Carlos (UFSCar), São Carlos, São Paulo 13565-905, Brazil; ⊥ Institute of Scientific and Technological Research in Electronics (ICYTE), University of Mar del Plata and National Research Council (CONICET), Mar del Plata B7608FDQ, Argentina; # Departamento de Química y Bioquímica, Facultad de Ciencias Exactas y Naturales, Instituto de Investigaciones Físicas de Mar del Plata (IFIMAR), CONICET, Universidad Nacional de Mar del Plata (UNMDP), Mar del Plata 7600, Argentina; ∇ Institute of Materials Science and Technology (INTEMA), National University of Mar del Plata and National Scientific and Technical Research Council (CONICET), Mar del Plata 7600, Argentina

## Abstract

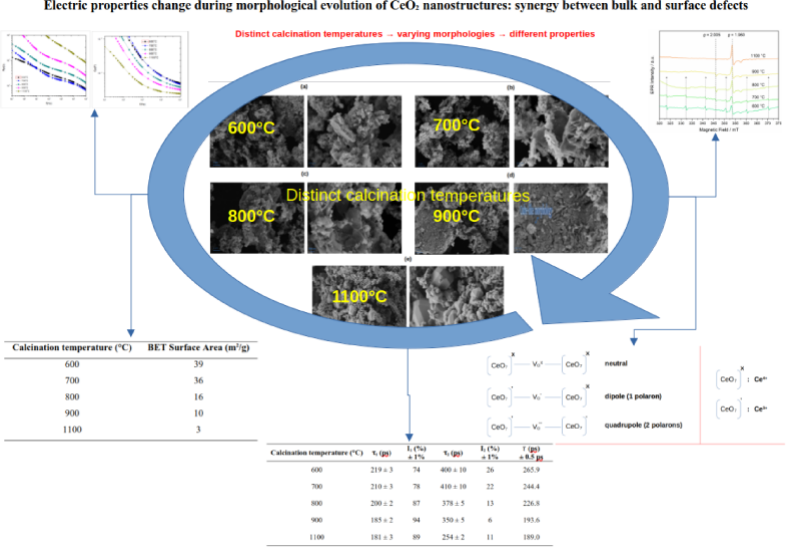

CeO_2_ samples were synthesized via the polymeric precursor method at different calcination temperatures. Electric properties were investigated using positron annihilation lifetime spectroscopy, electron paramagnetic resonance spectroscopy, and complex impedance spectroscopy. Reduction in the specific surface area of the particles with increasing calcination temperature along with morphological changes were observed. PALS depicted doubly ionized oxygen vacancies surrounded by two Ce3+ atoms, while the EPR spectroscopy showed a singly ionized oxygen vacancy surrounded by Ce3+ and Ce4+ ions. Impedance measurements unraveled the presence of polarons, while thermal treatments led to a lower electrical conductance, as the calcination temperature increased.

## 1 Introduction

Cerium dioxide (CeO_2_) is a versatile reducible oxide widely used in industrial processes, including the neutralization of engine exhaust gases^1^ and the partial oxidation of methane and degradation of phenol in wastewater due to its wide accessibility, lack of toxicity, and excellent stability.^2,3^ Due to its unique properties (i.e., high oxygen storage capacity (OSC) and high oxygen mobility stemming from the Ce^4+^/Ce^3+^ redox pair,^4,5^ CeO_2_ stands out as an important multifunctional rare earth oxide in catalytic applications. Catalysis is a widespread process in manufacturing, with more than 60% of manufactured products undergoing at least one catalytic cycle.^6^ In this context, Cargnello et al. showed that CeO_2_ plays a pivotal role as a support material in carbon monoxide (CO) oxidation processes.^7^ Besides, Vayssilov G. N. et al.^8^ demonstrated two types of oxidative metal-oxide interaction on Pt-ceria catalysts, showing that electrons are transferred from the Pt nanoparticles to the support along with oxygen transfer from ceria to Pt, with the electron transfer occurring irrespective to their morphology, corroborating the extraordinary structure–activity dependence of ceria-based catalysts. In this sense, thermal treatments that change the overall structure are also of importance to this matter. Additionally, Varga, E. et al.^9^ studied the interaction of CeO_2_-supported Rh, Co and bimetallic Rh–Co nanoparticles, which are active catalysts in hydrogen production via steam reforming of ethanol and identified similar features of electron transfer from Rh to ceria and the oxygen transfer from ceria to Rh.

On the other hand, the sensing properties of CeO_2_ toward CO have been extensively investigated ,^10−14^ and confirm its improved sensory response to CO in comparison with SnO_2_. The interaction between CO molecules and adsorbed oxygen/lattice oxygen at the surface of CeO_2_ increases its electrical conductivity due to the increased oxygen vacancy concentration in the material, which also results in the reduction of Ce^4+^ to Ce^3+^.^14^

When studying rare-earth doped CeO_2_ nanoparticles synthesized via the microwave-assisted hydrothermal (MAH) method, Ortega et al.^13,15^ reported that oxygen vacancies were the dominant defect and the fastest response time to CO was obtained for Eu-doped nanoparticles, while Rocha et al.^16^ observed a dual response (optical and electrical) of La-doped CeO_2_ nanoparticles toward CO. Oliveira et al.^17^ studied the influence of the synthesis time on the morphology of ceria nanostructures and proposed that the predominant growth mechanism were Ostwald ripening, together with oriented attachment. Amoresi et al.^18^ obtained distinct ceria nanostructures (cubes, rods, hexagons, and beans) by changing the mineralizer concentration and then tested their photocatalytic activity against the ciprofloxacin antibiotic. The authors reported that different functional groups of the antibiotic molecules were attacked depending on the morphology of the CeO_2_ nanostructure. In short, the literature has shown that the properties of materials depend on their structures and morphologies. In this context, thermal treatments emerge as a promising strategy for inducing the crystallization of different morphologies with distinct properties, as demonstrated for other oxides showing the same behavior.^19,20^

The electric properties of ceria-based materials are predominantly controlled by hopping conduction^21^ and the small polaron theory. The charge conduction process is dominated by the cluster-to-cluster charge transfer (CCCT) mechanism,^22^ with its probability depending on the distance between adjacent sites. Blumenthal et al.^23,24^ reported that the reduced state of ceria (CeO_2–x_) is a necessary condition to allow hopping conduction, which has an activation energy (Ea) of 0.22 eV for small x, gradually increasing as x increases. The hopping process depends on the carrier density (4f^1^ electrons of Ce^3+^), which is, in turn, correlated with the electrons trapped in the Ce^3+^/Ce^4+^ redox pairs. This pair forms when one of the electrons left from an oxygen atom during the vacancy generation is transferred to a Ce^4+^ atom, resulting in a Ce^3+^ surrounded by eight oxygen atoms, the so-called [CeO_8_]’ quantum cluster.^22^ If one electron remains trapped in a vacancy, a singly positive ionized vacancy is formed, i.e., [Ce_Ce_]’-V_O_**^˙^**-[Ce_Ce_]^x^. On the other hand, if two electrons leave the neutral oxygen vacancy, Vo, a doubly ionized vacancy is formed, i.e., ([Ce_Ce_]’- V_O_¨- [Ce_Ce_]’). These electrons can remain in the quantum cluster surrounding the vacancy or become an electrical carrier and generate a [CeO_8_]’ polaron far from the originating vacancy. This dipole type was proposed by Lu Sun et al. in their theoretical studies^25^ and can be tracked using the EPR technique. In the same work, it was also demonstrated that the presence of [Ce_Ce_]’ – V_O_¨- [Ce_Ce_]’ quadrupoles can be tracked using the PALS technique, corroborating the findings reported by Sudarshan et al.^26^

**Scheme 1:**
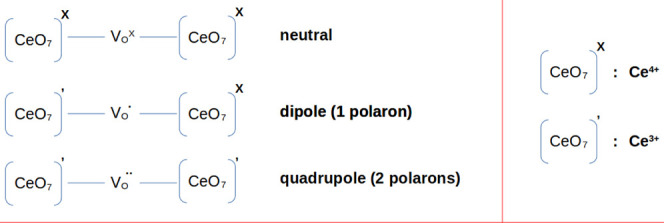
Schematic Representation of Neutral, Singly, and Doubly Ionized Oxygen Vacancies as Clusters Containing Polarons

At this point, it is worth mentioning that excess electrons can originate from donor impurities or intrinsic defects, such as oxygen vacancies, and that they tend to localize in Ce atoms, forming Ce^3+^ species and resulting in a local lattice distortion, slightly elongating the Ce^3+^-O bonds. Electron localization and local lattice distortion combined lead to the formation of small polarons, which can be used to represent defective clusters present in ceria, as depicted in Scheme 1.

In the present work, we propose that positrons annihilated in small neutral complexes formed by a positive oxygen vacancy (V_O_¨) and two negative ions are located around the vacancy.^27,28^ Additionally, in order to determine the role of different oxygen vacancy complexes (OVCs) in the electrical conduction of the materials, thermal treatments (TT) were carried out at 600, 700, 800, 900, and 1100 °C. Particularly, attention is given to the hopping conduction mechanism resulting from the presence of different species in cerium oxide.

## 2 Experimental Section

The CeO_2_ samples were synthesized via the polymeric precursor method (PPM). In the first step, cerium nitrate hexahydrate and citric acid were separately dissolved in deionized water. After homogenization, the citric acid solution was added to the cerium precursor solution under constant stirring at 60 °C for 30 min (Ce complexation step). Next, ethylene glycol was added to the mixture and stirred for 12 h at 50 °C (polyesterification step). The Ce metal: citric acid:ethylene glycol stoichiometric ratio was 1 mol Ce:4 mol citric acid:16 mol ethylene glycol. 400 mL of the Ce polymeric resin was kept under stirring at 110 °C for 3 weeks. After the polyesterification step, the resin was precalcinated at 300 °C for 120 min at a heating rate of 5 °C/min. The resultant material was then macerated and sieved on a 53 μm sieve (270 mesh). Finally, the precalcinated powder was heat-treated in alumina crucibles at 600, 700, 800, 900, and 1100 °C for 120 min (5 °C/min heating rate), after which fine light-yellow CeO_2_ nanoparticles were obtained.

The samples were characterized using positron annihilation lifetime spectroscopy (PALS), electron paramagnetic resonance spectroscopy (EPRS), complex impedance spectroscopy (CIS), specific surface area (BET) measurements, and scanning electron microscopy (SEM). To determine the surface area of the different CeO_2_ samples, a Micromeritics FlowSorb II 2300 surface area analyzer was used. All areas were obtained from one-point adsorption isotherms using N_2_ at −196 °C as an absorbate. To this end, approximately 200 mg of each sample (previously dried in an oven at 120 °C) was transferred to a glass sample holder, which to completely dry the sample, was placed in the equipment position at 250 °C in an N_2_ atmosphere for the desorption of water and impurities. The N_2_ atmosphere, which was consistently used throughout the experiment, was adsorbed at −196 °C by immersing the sample holder in a Dewar flask with liquid N_2_, and the adsorbed N_2_ (desorption) after removing the sample holder from the Dewar flask was measured to determine the surface areas. The method used for the measurements was Brunauer-Emmet-Teller (BET). The scanning electron microscopy images were acquired on a FEG-SEM Supra 35-VP microscope at an accelerating voltage of 5 kV. The PALS was performed in a fast–fast system instrument with a collinear geometry and 275 ps time resolution. A^22^Na deposited between two Kapton foils (7.5 μm thick each) was used as the positron source, which was encased between two identical samples. The measurements were carried out at room temperature, and usually 1.5–2 × 10^6^ counts per spectrum are collected. The reported lifetime values were averaged ten times in the same experimental conditions. The spectra were analyzed using the LT10 code after background and source subtractions.^29^ The EPR spectroscopy was performed at 293 K using an ELEXSYS E500T instrument (Bruker, Germany), provided with an ER 4102ST rectangular resonator in the TE102 mode at a center frequency of 9.8 GHz (X-band). The acquisition parameters used were: 100.00 kHz modulation frequency, 0.40 mT modulation amplitude, 20.4 mW microwave power, 347.5 mT central field, 55 mT sweep width, and 20.48 ms conversion time. Each scan consists of a total of 2048 data points, and the final spectra were averaged ten times. For the electrical measurements, the powders were pressed to form disks with a diameter of 8 mm and a thickness of 1.5 mm. The CIS measurements were performed on a 3522–50 LCR HiTESTER (HIOKI), with the samples being placed in a 9263 SMD TEST FIXTURE (HIOKI) sample holder. The measurements were performed in air at room temperature at a frequency range of 10^–2^–10^5^ Hz.

## 3 Results and Discussion

Worth mentioning that these samples were obtained in a previous study^30^ and their phase characterization corroborated the formation of crystalline CeO_2_ particles.

### 3.1 Specific Surface Area (BET)

Table 1 presents the specific surface area measurements for the samples calcined at distinct temperatures, where an overall reduction can be observed with increasing temperature. These results agree with those obtained for ceria nanostructures by Yang et al.,^31^ who reported values ranging from 56.3 to 3.3 m^2^/g as the temperature increased from 500 to 800 °C. Despite counterintuitive, the authors reported an increased photodegradation rate of methylene blue under UV irradiation from 67 to 98% for the samples calcined at 500 and 800 °C, respectively. They concluded that the specific surface area was not the determining parameter associated with the photocatalytic efficiency, with the calcination temperature playing a significant role.

**Table 1:** Specific Surface Area Measurements for the Ceria nanostructures Calcined at Distinct Temperatures

Calcination temperature (°C)	BET Surface Area(m^2^/g)
600	39
700	36
800	16
900	10
1100	3

It is important to note that owing to the quantum character of the electrical conduction mechanism in ceria-based nanomaterials substantiated by the small polaron theory,^32-34^ the reduction in specific surface area is not always related to a reduction in the reactivity, as depicted by Rocha et al.^16^ when studying pure and La-doped CeO_2_ nanoparticles. In this case, the doped sample showed a reduced specific surface area in comparison with the pure sample (86 m^2^/g against 120 m^2^/g), despite a faster response time for CO_(g)_ detection (5.5 against 54 s, respectively).^22^ Therefore, the electron transfer process, known as cluster-to-cluster charge transfer (CCCT), has a probability that roughly depends on the distance of neighboring species containing oxygen vacancies and Ce(III)/Ce(IV) pairs^17^ and is locally controlled by 4f^1^ states, which would explain the opposing characteristics of reduced specific surface area and improved reactivity. Regarding the catalytic activity of CeO_2_ for CO oxidation, Guo et al.^35^ found that the increase in calcination temperature from 200 to 800 °C gradually decreased the specific surface area of the sample from 457 to 21 m^2^/g, as well as its catalytic activity. Therefore, it is safe to say that when dealing with nanostructured materials, several factors such as surface area, particle size, morphology, and number of defective species play a key role, with a delicate balance, in these materials’ properties, as also stated by Trovarelli,^34^ and that various spectroscopic and structural features must be considered for a proper characterization of their behavior.

### 3.2 Scanning Electron Microscopy (FEG-SEM)

**Figure 1:**
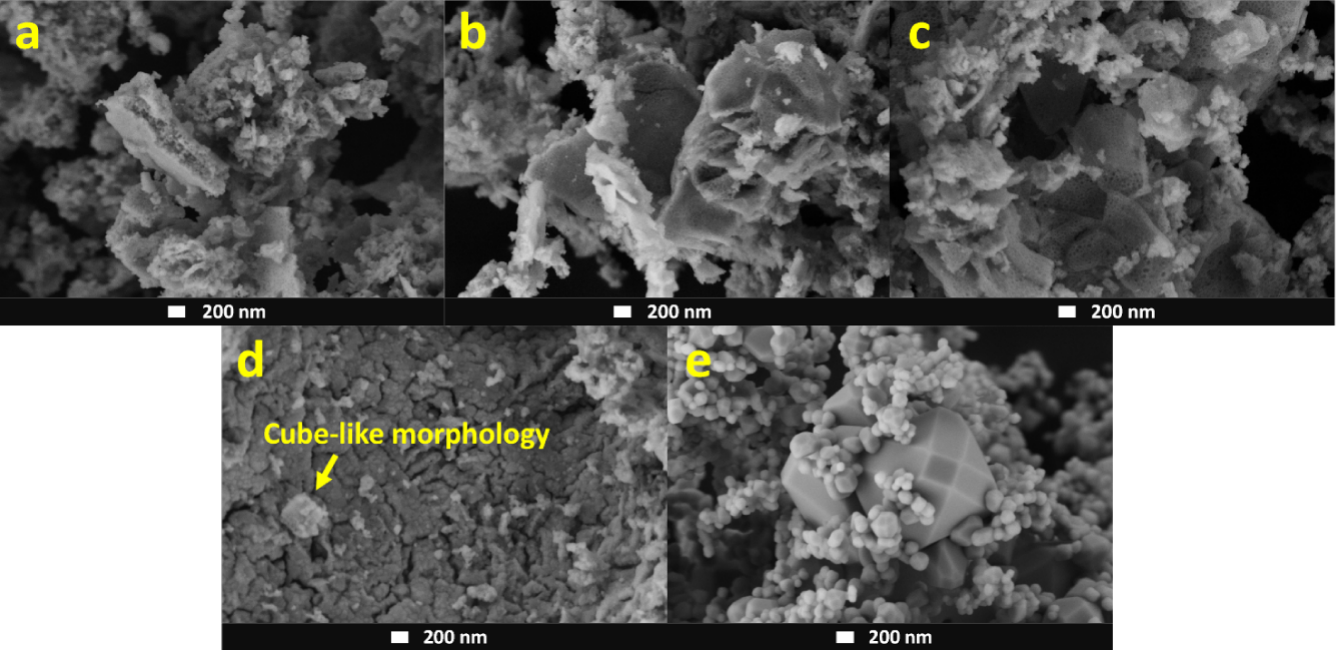
Scanning electron microscopy images of the CeO_2_ samples calcined at 600 (a), 700 (b), 800 (c), 900 (d), and 1100 °C (e).

Figure 1 shows the scanning electron microscopy images of the samples after calcination from 600 to 1100 °C, revealing an interesting feature regarding their morphological behavior. At the first calcination temperature, the system exhibits agglomerated particles with structures surpassing the 200 nm scale but with no well-defined morphologies (Figure 1a). When the calcination temperature is increased to 700 °C (Figure 1b), the agglomerated systems start to develop sheet-like morphologies with an apparent porosity, which then expand into three-dimensional structures, as observed for the system calcined at 800 °C (Figure 1c), and forming cubic particles, as indicated in the sample calcined at 900 °C (Figure 1d). At higher calcination temperatures, the particles start to develop well-defined octagon-like morphologies, as clearly seen in the sample heat-treated at 1100 °C (Figure 1e).

Ho et al.^36^ prepared distinct shapes of CeO_2_ nanoparticles by mixing (NH_4_)_2_Ce(NO_3_)_6_ and ethylene glycol and varying both the cerium precursor concentration and the reaction duration. Briefly, spheres were obtained at a cerium precursor concentration of 80 mM and a reaction time of 4 h, while rod-like particles were formed at the same precursor concentration but increasing the synthesis time to 24 h. Spindle-like morphologies were observed at the same synthesis time but reducing the concentration to 40 mM. Besides, the authors concluded that the CeO_2_ nanospheres were built up from the aggregation of small crystallites, as clearly seen in our samples.

We can also cite other studies addressing the influence of calcination temperature on the morphology of ceria particles. For instance, rod-like particles with an average length of 7 μm were obtained by the conventional hydrothermal route after 1 h of calcination from 300 to 900 °C,^37^ whereas flower-like morphologies developed after calcination at 500 °C for 5 h and 800 °C for 2 h and hexagonal shapes and microplates with 3.8–6.0 μm in length and 0.8 μm in thickness were obtained by the same route, but at a postsynthesis calcination temperature of 500 °C for 1 h.^38^

Therefore, it can be inferred that highly crystalline CeO_2_ microstructures can be achieved with different morphologies, with the reaction time and surfactant concentration being significant factors that affect the structural evolution of ceria-based nanomaterials. In this context, postsynthesis heat treatments are considered an effective strategy to tune the properties of these materials for specific applications. Very broad particle size distributions (nanoparticles) lead to grain growth, causing agglomerates to densify first. Then, large pores collapse in the first minutes of densification owing to particle asymmetries, leading to a high rate of densification. Calcination atmospheres also play an important role in both densification and pore evolution, being directly related to vacancy density.

### 3.3 Positron Annihilation Lifetime Spectroscopy (PALS)

PALS is a robust, nondestructive, sensitive, and versatile technique used to investigate the defect structure of nanostructures, which encompasses the type and concentration of defects present at the nanoscale in metals and semiconductors.^39^ Usually, multiple lifetime components with specific lifetime values and associated intensities can be extracted from the decomposition of PALS spectra. In crystalline systems, the existence of distinct lifetime components is associated with the trapping and annihilation of positrons in various types of vacancy-like defects, including vacancies, dislocations, and grain boundaries. The positron lifetime value characterizes the type of defect, while its associated intensity is related to the defect concentration. Conversely, when defects are absent, the measured positron lifetime is referred to as bulk lifetime (τ_b_), which is characteristic of each material and acts as a reference for detecting the presence of defects.

In this work, all measured PALS spectra were well-fitted, considering three lifetime components. The longest lifetime component, approximately 2 ns, exhibited a minimal associated intensity (<1%). As this component is commonly associated with positron annihilation at the air-sample interface and thus does not provide pertinent physical information about the samples, it will not be considered from now on. Table 2 presents the positron lifetime and intensity parameters derived from the decomposition of the PALS spectra for the CeO_2_ samples calcinated at different temperatures. Additionally, the mean positron lifetime (), calculated from the individual lifetimes and intensities given by , is also shown in Table 2. Such a parameter, which is the most robust statistical parameter, represents the signature of the total electron density in the material.

**Table 2:** Characteristic Positron Lifetimes, Associated Intensities, and Mean Positron Lifetime Obtained from the PALS Spectra Decomposition for Samples Calcined at Distinct Temperatures

Calcination temperature (°C)	τ_1_(ps)	I_1_(%) ± 1%	τ_2_(ps)	I_2_(%) ± 1%	τ̅(ps) ± 0.5 ps
600	219 ± 3	74	400 ± 10	26	265.9
700	210 ± 3	78	410 ± 10	22	244.4
800	200 ± 2	87	378 ± 5	13	226.8
900	185 ± 2	94	350 ± 5	6	193.6
1100	181 ± 3	89	254 ± 2	11	189.0

As a general feature, in Table 2 it can be seen that  decreases with increasing calcination temperature, indicating an overall decrease in the number of defects in the material as the calcination temperature increases.

Considering the individual components, it is possible to observe that for calcination temperatures between 600 and 900 °C, the τ_1_ values decrease from ∼219 ps up to ∼185 ps, while their associated intensity (I_1_) increases from about 74% to approximately 94%. On the other hand, the τ_2_ values range from ∼400 ps for the sample calcined at 600 °C, slightly increasing within the experimental error for the sample calcined at 700 °C and decreasing to 350 ps for the one calcined at 900 °C. As for the I_2_ values, they systematically decrease from ∼26% to approximately 6% with increasing calcination temperature. Regarding the sample annealed at 1100 °C, τ_1_ values of ∼181 and ∼254 ps with associated intensities of ∼89% and ∼11%, respectively, were obtained. Given the change in trend observed in the results obtained for the sample annealed at 1100 °C, it can be concluded that they should be analyzed separately from those obtained for the samples calcined between 600 and 900 °C.

According to the literature, when studying different nanoparticulate systems such as nanocrystalline oxides and metal-based materials using PALS measurements, one can obtain typical values between approximately 350 and 500 ps for the second temporal component.^40−45^ These t_2_ values are generally attributed to the trapping and subsequent annihilation of positrons in defects with a large open volume, such as vacancy clusters located on the surface of nanograins or in intergranular spaces, namely, intergranular defects. In these cases, the obtained value is directly related to the extended defect volume.^39^ Based on the τ_2_ values found for the samples calcined from 600 to 900 °C in this study, it is reasonable to assume that the second temporal component obtained in all analyzed samples can be attributed to Ce^3+^-coordinated positrons trapped and annihilated in oxygen vacancy complexes (OVCs) both on the material surface and subsurface, as previously reported by Chang et al.^43^ and Shi et al.^46^ In contrast, in the mentioned papers,^44,45^ the shorter temporal component is ascribed to positrons annihilated in small neutral associations of Ce^3+^ oxygen vacancies located inside the nanograins. It is important to mention that the effectiveness of this type of defect in trapping positrons depends on the total charge of the vacancy defect complex.

Concerning the results obtained for the sample calcined at 1100 °C, the τ_2_ value (τ_2_ = 254 ps) is typical of those reported for positrons annihilated in monovacancies in solids. According to the literature, a positron lifetime between 200 and 260 ps can be attributed to the annihilation of positrons in small neutral associations of Ce^3+^ oxygen vacancies,^46^ that is, [Ce_Ce_]’- V_O_¨- [Ce_Ce_]’ quadrupoles consisting of doubly ionized oxygen vacancies (V_O_¨) surrounded by two Ce^3+^ ions ([Ce_Ce_]’). Similarly, a positron lifetime of 253 ps was reported for oxygen vacancies in Gd-doped ceria.^47^

In this scenario, the obtained τ_1_ values for the samples calcined from 600 to 900 °C are higher than that reported in the literature for defect-free ceria (τ_b_ = 187 ps),^44^ but smaller than that found for quadrupoles (τ_2_ = 254 ps). Consequently, our τ_1_ values reveal that positrons annihilate in a mixed state containing defect-free ceria and quadrupoles. Additionally, these values systematically decrease as the quadrupole concentration decreases with increasing calcination temperature. In this way, sintering eliminates structural defects and oxygen vacancies, increasing symmetric quantum clusters and reducing disorder in the semiconductor. Therefore, there is a variation in the conductivity of the material.

To unravel these two contributions to the τ_1_ component and obtain values for the concentration of intragranular vacancies, the data presented in Table 2 were analyzed using the positron diffusion trapping model.^48^ This model proposes the competitive trapping of positrons in intragranular point defects and at grain boundaries of polycrystalline materials under the assumption of spherical grains. The lifetime values of positrons annihilated before being trapped (τ_0_), the fraction of positrons trapped by quadrupoles (I_v_), the trapping rate in vacancies (*κ*_*v*_), and the trapping rate at grain boundaries (*κ*_*GB*_) were calculated by solving numerically the following set of equations (for more details, see ref.^48^
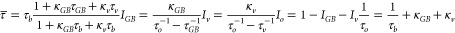
1under the following assumptions: (a) the modeled physical situation corresponds to the limiting case of high positron diffusion and/or small grain size; (b) the lifetime value corresponding to positrons trapped by quadrupoles, τ_v_ = 254 ps, is the same for all studied samples; and (c) the lifetime values of intergranular defects (τ_GB_) and their corresponding intensity (I_GB_) are those corresponding to τ_2_ and I_2_ in Table 2. The obtained results can be seen in Table 3. It is important to point out that *κ*_*v*_ is directly proportional to the defect concentration.^39^ Specifically, the positron trapping rate can be defined as *κ*_*v*_*=μ*_*v*_*C*_*v*_, where *μ*_*v*_ is the defect-specific trapping rate, and *C*_*v*_ is the defect concentration – [Ce_Ce_]’- V_O_¨-[Ce_Ce_]’ quadrupoles, in our case.

**Table 3:** Lifetime Values of Positrons Annihilated before Trapping (τ_0_), Positrons Trapped by [Ce_Ce_]’- V_O_¨-[Ce_Ce_]’ Quadrupoles (τ_v_) and their Corresponding Intensity (I_v_), Positrons Trapped by OVCs (τ_GB_) and Their Corresponding Intensity (I_GB_), Trapping Rate in Vacancies (*κ*_*v*_), and Trapping Rate at Grain Boundaries (*κ*_*GB*_) Calculated Solving the Set of eq 1

Calcination temperature (**°**C)	τ_0_(ps)	τ_v_(ps)	I_v_(%)	τ_GB_(ps)	I_GB_(%)	*κ*_*v*_ (×10^9^*S*)	*κ*_*GB*_**(×10**^**9**^*S*)
600	58	254	61	400	26	8.1 ± 0.9	3.9 ± 0.5
700	100	254	56	410	22	3.4 ± 0.5	1.7 ± 0.2
800	108	254	55	378	13	2.9 ± 0.3	0.90 ± 0.05
900	159	254	27	350	6	0.66 ± 0.05	0.19 ± 0.02
1100	181	254	11	-	0	0.18 ± 0.02	0

**Figure 2:**
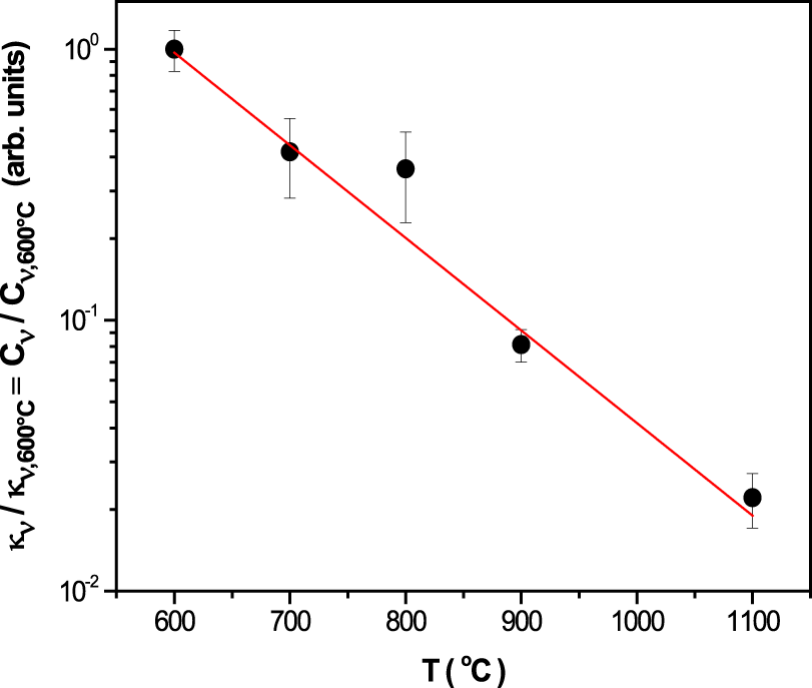
Evolution of [CeCe]’- VÖ-[CeCe]’quadrupole concentration in relation to that obtained for the sample calcinated at 600 °C as a function of the calcination temperature. The solid line is only an eye guide.

As seen in Table 3, from the behavior of I_v_ and *κ*_*v*_, the concentration of intragranular quadrupoles systematically and significantly decreases as the calcination temperature increases. The ratio of the positron trapping rate in vacancies, *κ*_*v*_, obtained for two different temperatures indicates a change in the concentration of quadrupoles. Figure 2 shows this change for the different calcination temperatures using those corresponding to the initial one (obtained at 600 °C). As it can be observed, approximately 2% of the quadrupoles present in the initial sample calcinated at 600 °C remain in the sample calcinated at 1100 °C.

Furthermore, from Table 3, it can be seen that τ_GB_ and I_GB_ values also systematically decrease as the calcination temperature rises. The behavior of τ_GB_ indicates that the OVCs located on the grain surfaces become smaller as the calcination temperature increases, suggesting that atmospheric O atoms become fixed in these vacancy clusters, having their size reduced. On the other hand, the trend shown by I_GB_ as a function of the calcination temperature suggests that as T increases, there is a progressively smaller number of OVCs on the surface of the nanograins until reaching a negligible amount for the sample calcinated at 1100 °C. It means that bigger nanograins, characterized by smaller specific surface areas would enable the formation of fewer OVCs as already reported in ref.^45^

### 3.4 Electron Paramagnetic Resonance Spectroscopy (EPRS)

EPR spectroscopy is a powerful tool for probing surface and core defects in metal oxide semiconductors, with particular relevance in catalytic and sensor materials.^49^ For instance, EPR has been employed to characterize intrinsic defect centers, such as oxygen and zinc vacancies, in ZnO nanocrystals milled under cryogenic conditions. Kaftelen et al.^50^ identified three distinct surface defects with varying g-factors, noting that their spectral intensities shifted with decreasing crystal size. Through complementary optical emission studies, the authors proposed a core–shell model that distinguishes between electronic states within the band gap. This model attributes negatively charged zinc vacancies to the core and positively charged oxygen vacancies to the shell. Similarly, EPR has proven to be a useful technique in examining single-component core–shell structured TiO_2_.^51^ By integrating EPR spectroscopy with X-ray photoelectron spectroscopy, Hu et al.^52^ investigated the electronic structure of core–shell structured TiO_2_. Their results revealed that the shell of TiO_2_ was predominantly composed of Ti^4+^, while the core retained a significant presence of Ti^3+^ ions. This conclusion was supported by the pronounced EPR signal attributed to Ti^3+^ centers, which were identified as regular Ti sites perturbed by the presence of oxygen vacancies. Similarly, Laguta et al.^53^ demonstrated the efficacy of EPR as a powerful analytical technique for investigating oxygen-vacancy-related defects in perovskite-type ferroelectric BaTiO_3_ thin films. The authors successfully identified Ti^3+^–V_O_ centers, highlighting the utility of EPR in providing detailed insights into the electronic and defect structure of BaTiO_3_, particularly in relation to the role of oxygen vacancies in influencing the material’s ferroelectric properties.

Regarding CeO_2_, significant advancements have also been made in the study of surface defects through EPR. A commonly employed technique involves subjecting CeO_2_ to a reductive outgassing pretreatment under carefully controlled conditions that promote the formation of surface defects, particularly Ce^3+^ cations associated with oxygen vacancies. These surface defects possess the unique ability to chemisorb oxygen molecules, resulting in the generation of superoxide species. The subsequent analysis of these superoxide species using EPR spectroscopy provides in-depth insights into the local structural environment and the redox characteristics of the material’s surface.^54^ Soria et al.^55^ studied the adsorption of oxygen, with either normal oxygen or ^17^O-enriched mixtures, on CeO_2_ that had been outgassed at varying temperatures. The EPR analysis revealed distinct signals corresponding to O_2_^–^ species bound to surface cerium ions, with the observed signals varying depending on the vacuum treatment temperature. The authors identified two primary types of O_2_^–^ signals, differentiated by their line widths and g values. These signals are associated with species adsorbed at isolated and aggregated oxygen vacancies, respectively. Importantly, the EPR parameters indicated that the bonding of these species to the surface exhibits varying degrees of covalency. For O_2_^–^ species formed at isolated surface oxygen vacancies, the two oxygen atoms are EPR equivalent, whereas, in species formed on aggregated vacancies, typically generated at higher outgassing temperatures, the oxygen atoms are nonequivalent and might be in some cases bonded to Ce^3+^ ions. The variations in EPR signal parameters and intensity, observed under different vacuum treatment conditions and subsequent thermal treatments, highlight the sensitivity of EPR in probing the nature and properties of surface defects on CeO_2_.

Numerous studies have applied the O_2_-probe EPR technique to investigate CeO_2_, as detailed in the scientific literature.^54,56−58^ While these investigations have significantly contributed to the understanding of surface oxygen vacancies in CeO_2_, the O_2_-probe EPR method requires a controlled outgassing and subsequent O_2_ absorption of the sample, coupled with low-temperature (≤77 K) EPR measurements. While the application of this technique is beyond the scope of our work, our study focuses specifically on paramagnetic species observable at room temperature, which also provide valuable insights into the defects present in the material, as discussed below.

**Figure 3:**
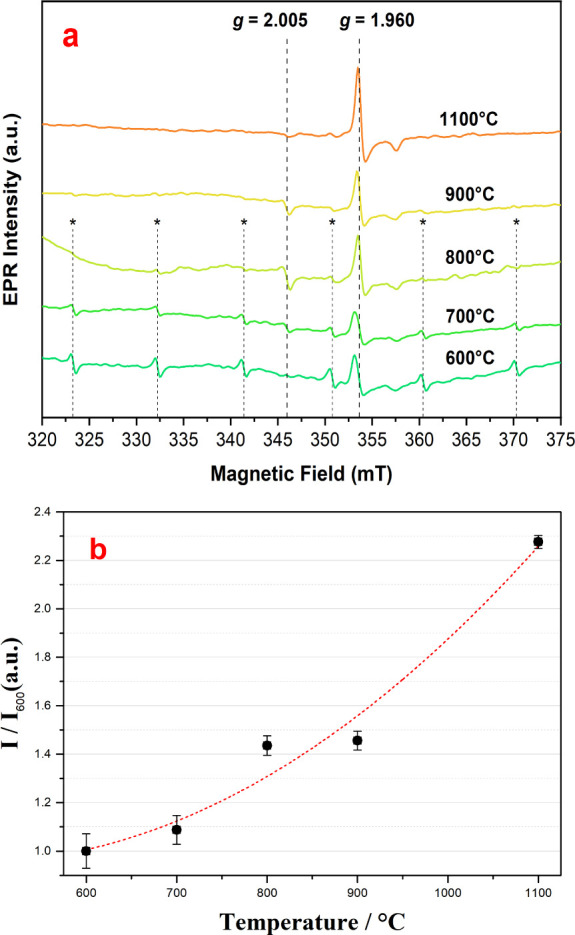
(a) EPR spectra of the CeO_2_ samples calcined at 600, 700, 800, 900, and 1100 °C. The sextuplet signal indicated with asterisks corresponds to the presence of Mn^2+^ as an impurity. (b) Rate of EPR signal for *g* = 1.960 vs. calcination temperature.

Figure 3 displays the EPR spectra of the CeO_2_ samples calcined at 600, 700, 800, 900, and 1100 °C. A sextuplet signal can be distinctly observed for the samples calcined at 600 and 700 °C, partially verified for the sample calcined at 800 °C, and undetected for the samples calcined at 900 and 1100 °C. This distinctive signal can be ascribed to the presence of residual Mn^2+^ impurities, which are rather common in CeO_2_ samples.^59-61^ As the calcination temperature increases, the intensity of the Mn^2+^ signal decreases because Mn^2+^ is oxidized to Mn^3+^, which can be EPR silent.

An EPR signal at a *g* factor of 2.005 was detected for the samples calcined at 700, 800, 900, and 1100 °C. In the latter, the signal intensity was low, while no signal was detected for the sample calcined at 600 °C. Similar signals with *g* factors very close to that of the free-electron were ascribed to unpaired electrons in oxygen vacancies^16^ and oxygen absorbed on the surface of CeO_2_.^17^ Another distinctive signal was observed at a *g* factor of ca. 1.96. Although this signal is characteristic of CeO_2_ samples, there is some controversy regarding its origin. In several scientific reports, this signal is assigned to paramagnetic Ce^3+^ species.^62−69^ However, it is known that Ce^3+^ is usually hard to detect at temperatures higher than 20 K due to the strong spin–orbit coupling that results in short relaxation times of 4f^1^ states.^70^ Furthermore, a *g* factor of ca. 1.96 is rather close to that of the free electron (*g* = 2.0023). Thus, it was expected that Ce^3+^ species would exhibit larger deviations from this value. In fact, *g* factors of *g*_⊥_ = 1.396 and *g*_∥_ = 3.038 were observed at 4.2 K for Ce^3+^-doped CaF_2_, which is isostructural with CeO_2_.^59^ Rakhmatullin et al.^71^ conducted an EPR study on CeO_2_ nanoparticles containing different concentrations of Ce^3+^. No correlation between the amount of Ce^3+^ present in the sample and the signal intensity at *g* ≈ 1.96 was observed, leading to the conclusion that the signal could not be assigned to Ce^3+^ species, but to electrons trapped near the surface of Ce^3+^/Ce^4+^ redox pairs. This pair is formed when one of the electrons left from an oxygen atom during a vacancy generation is transferred to a Ce^4+^ ion, while the other electron remains trapped in the vacancy void [Ce_Ce_]’-V_O_**^˙^**-[Ce_Ce_]^x^.^71^ According to Figure 3b, the intensity of the EPR signal increases as a function of calcination temperature. It can then be inferred that the samples calcined at higher temperatures exhibit a higher number of [Ce_Ce_]’-V_O_**^˙^**-[Ce_Ce_]^x^ species resulting from dipole formation, accompanied by a reduced specific surface area, as seen in the B.E.T measurements. In short, the PALS measurements show a strong decrease in [Ce_Ce_]’- V_O_¨-[Ce_Ce_]’, while the EPR analysis point to a slight increase in [Ce_Ce_]’-V_O_**^˙^**-[Ce_Ce_]^x^. On the other hand, the increase in the crystalline symmetry of ceria directly influences the Coulomb interactions (medium range) with a greater organization at the spin level (short-range).

### 3.5 Complex Impedance Spectroscopy (CIS)

**Figure 4:**
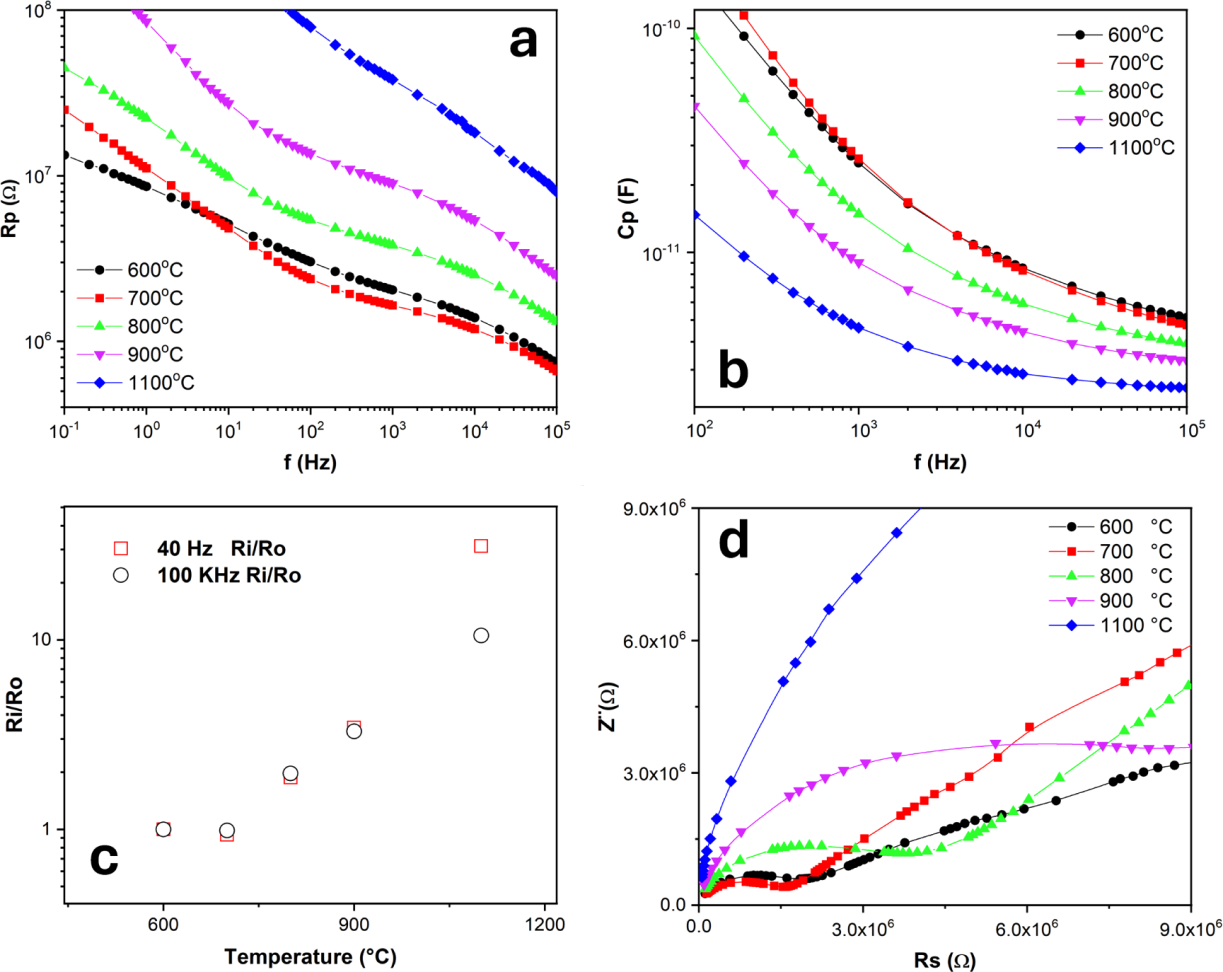
Complex impedance spectroscopy measurements for the CeO_2_ samples calcined at distinct temperatures. (a) Rp (Ω) vs f (Hz); (b) Cp (Ω) vs f (Hz); (c) Ri/R_0_ vs temperature (°C); and (d) Nyquist plots for all thermal treatments.

The two-point probe CIS measurements were performed to study the grain, grain-boundary, and total electric conductivity of the ceria samples calcined at distinct temperatures. The typical impedance plots in Figures 4a–d show the parallel resistance (a) and capacitance (b) of the samples as a function of frequency (Hz), in addition to a magnification of Ri/R_600 °C_ for the higher (c) and lower (d) frequency regimens as a function of temperature.

Initially, it can be verified that the resistance increases as a function of calcination temperature. It is interesting to note that the BET measurements reveal an overall decrease in the specific surface area of the samples, which suggests an increase in particle size along with an increase in their resistance.^72^ Moreover, the resistance (Rp) curves have distinct slopes depending on the frequency, indicating the presence of at least two distinct conduction processes. Conduction processes are strongly dependent on the band gap, dipoles, and quadrupoles. In this way, there is a variation in electron density in the short and medium range, which results in changes in the conduction process.

On the other hand, at a higher frequency range, the sample calcined at 1100 °C exhibits a lower capacitance due to the presence of large particles that facilitate the charge transfer processes throughout the crystal, besides well-defined morphologies, as seen in Figure 1. According to the literature, there is a direct correlation between capacitance and permittivity.^73^Figure 4b shows the decreased electrical capacitance of the samples for the entire range of measured frequencies. With regards to the frequency-dependent capacitance, the observed behavior suggests the possible presence of multitraps corresponding to the species seen in Scheme 1, with a 1/f^2^ (Hz) dependence of Cp. The inflection point corresponds to the “corner frequency”,^74^ suggesting a disordered deep bulk trap location. These multitraps and their association are considered responsible for the modification of the Debye-like response.

Deep traps are activated by temperature, as evidenced by the dependence of the square of frequency. Contrarily, higher calcination temperatures decrease the effect of deep and shallow traps with increasing frequency. Additionally, at high frequencies, virtually no capacitance dependence on frequency is seen for all samples. This behavior is corroborated by the absence of band bending, or the fact that the band bending does not affect the sample capacitance.

**Figure 5:**
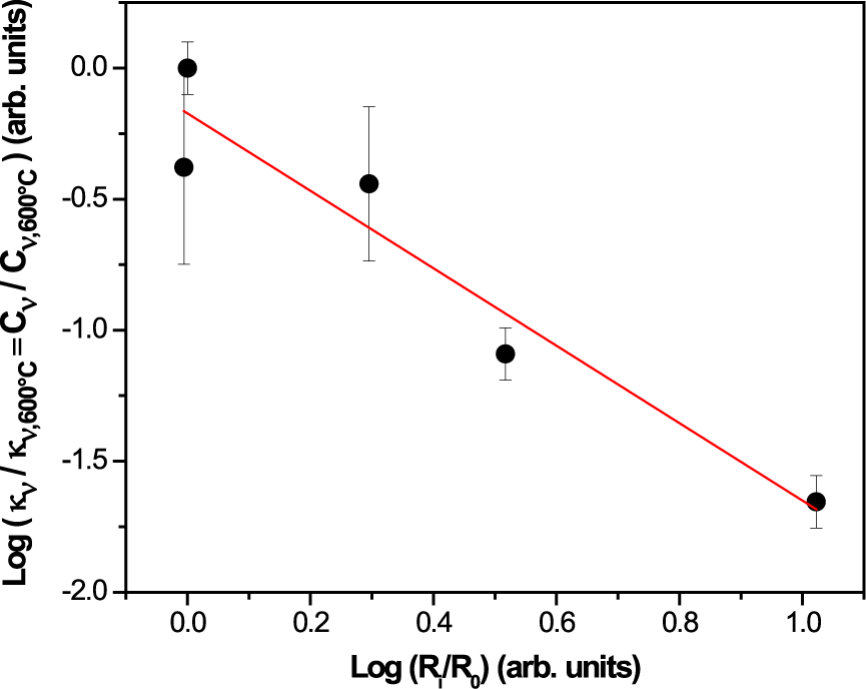
Linear fit of PALS vs IS.

A strong resistance dependence on frequency can be verified for all samples. Under the experimental conditions in which the samples were exposed to atmospheric air at room temperature, a variation in the hopping conduction would be a consequence of the calcination temperature modification, with the hopping tunneling distance increasing as a function of temperature^75^ due to the increased particle size and reduced specific surface area, which would justify the increase in resistance, as seen in Figure 4a. The increase in calcination temperature induces a decrease in the concentration of oxygen vacancies, as suggested by the literature.^75^ Consequently, the annihilation of oxygen vacancies reduces the number of charge carriers and thereby the ability to store charges, which would explain the decreased capacitance of the samples as the calcination temperature increases.^76^ This explanation is also valid for the interpretation of the increased resistance observed for the samples calcined at higher temperatures. According to the PALS analysis, the concentration of oxygen vacancies gradually decreased as the calcination temperature increased. As explained before, the formation of an oxygen vacancy is accompanied by the localization of electrons in Ce 4f states. Since the hopping conduction occurs via the hopping of electrons in those states, a decrease in the number of oxygen vacancies would also decrease the number of charge carriers, increasing the resistance, a trend that was clearly observed in Figure 5a.

The Nyquist plots in Figure 4d reveal the absence of electrode arcs, suggesting a predominance of electron conduction,^77^ as evidenced by the huge tails. Besides, as the calcination temperature increases, there is a trend toward an increase in the radius of semicircle arcs, except for the sample calcined at 600 °C, which seems to be out of this trend. When studying LSFC/Gd:CeO_2_ composite cathodes, Tomov et al.^78^ detected the presence of two overlapping arcs, in contrast to our study, which implies that the high-frequency arc is related to the charge transfer reaction at the LSCF:CGO interface, while the low-frequency arc is assigned to an oxygen surface exchange and/or dissociative oxygen absorption. In our case, the oxygen surface exchange and/or dissociative oxygen absorption can be attributed to the presence of a low-frequency arc observed in the samples calcined from 600 to 1100 °C.

In order to clarify the impedance spectroscopy results and their correlation with the evolution of quadrupoles, Figure 5 illustrates the linear fit of PALS vs IS response at 40 Hz. Since the EPR signal decreases as the sample conductivity increases (Figure 3b), we are impelled to ascribe the observed sample conductance to electrons due to the presence of doubly ionized oxygen vacancies detected in the PALS analysis.

The PALS signal intensity can then be attributed to the presence of native defects, more specifically oxygen vacancies, within the species with two polarons since they are neutral (see Scheme 1). This species behaves as a donor that, after ionization, donates two electrons for electrical conduction, as given by eqs 2 and 3:
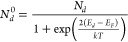
2
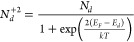
3where N_d_ is the concentration of total donors, N_d_^0^ is the concentration of neutral donors, N_d_^+2^ is the concentration of ionized donors, E_d_ is the donor level energy, and E_F_ is the Fermi level energy.

Thus, from eqs 2 and 3 we can write eq 3:
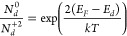
4

The concentration of N_d_^0^ is directly related to the PALS signal intensity, while N_d_^+2^ corresponds to the density of carriers at the Ce 4f levels, therefore being directly associated with the sample conductance. Finally, since the density of carriers exponentially depends on the Fermi level, eq 5 was proposed as follows:
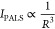
5

Such equation is reflected on a double logarithmic scale, as a straight line with slope −3. Figure 5 shows the experimental results, which are close to those obtained in this analysis.

## 4 Conclusions

In this work, we showed that the combination of PALS, EPR, and impedance spectroscopies can establish the presence of the following neutral complexes: [Ce_Ce_]’- V_O_¨-[Ce_Ce_]’ quadrupoles, [Ce_Ce_]’- V_O_˙ -[Ce_Ce_]^x^ dipoles, and [Ce_Ce_]^x^- V_O_¨-[Ce_Ce_]^x^ doubly ionized complexes. The PALS technique indicated the presence of doubly ionized oxygen vacancies surrounded by two Ce^3+^ atoms, that is, quadrupoles. Despite the fact that [Ce_Ce_]’- V_O_˙ -[Ce_Ce_]^x^ complexes increase as a function of the calcination temperature, they constitute a small percentage of total species and have a minor contribution to the electrical conductance of the material. Additionally, the calcination temperature is responsible for controlling the oxygen vacancy density in an inverse relationship, i.e., the higher the calcination temperature, the lower the concentration of [Ce_Ce_]’- V_O_¨-[Ce_Ce_]’ neutral complexes and the greater the crystallite sizes, in accordance with PALS and BET area measurements. Furthermore, the impedance spectroscopy measurements were consistent with the PALS analysis (which pointed to a smaller number of [Ce_Ce_]’- V_O_¨-[Ce_Ce_]’ neutral complexes), as they indicated a decreasing Fermi level energy with respect to the 4f cerium states. The lowest Fermi level position was directly linked to a smaller number of carriers and, therefore, a lower electrical conductance with increasing calcination temperature.
